# A new species of terrestrial frog of the genus *Noblella* Barbour, 1930 (Amphibia: Strabomantidae) from the Llanganates-Sangay Ecological Corridor, Tungurahua, Ecuador

**DOI:** 10.7717/peerj.7405

**Published:** 2019-08-12

**Authors:** Juan Pablo Reyes-Puig, Carolina Reyes-Puig, Santiago Ron, Jhael A. Ortega, Juan M. Guayasamin, Mindee Goodrum, Fausto Recalde, Jose J. Vieira, Claudia Koch, Mario H. Yánez-Muñoz

**Affiliations:** 1 Fundación EcoMinga, Fundación Oscar Efren Reyes, Baños, Tungurahua, Ecuador; 2 Unidad de Investigación, Instituto Nacional de Biodiversidad, Quito, Pichincha, Ecuador; 3 Instituto de Zoología Terrestre & Museo de Zoología, Instituto BIOSFERA, Colegio de Ciencias Biológicas y Ambientales COCIBA, Universidad San Francisco de Quito, Campus Cumbayá, Quito, Pichincha, Ecuador; 4 Museo de Zoología QCAZ, Pontificia Universidad Católica del Ecuador, Quito, Pichincha, Ecuador; 5 Laboratorio de Biología Evolutiva, Instituto BIOSFERA, Colegio de Ciencias Biológicas y Ambientales COCIBA, Universidad San Francisco de Quito, Campus Cumbayá, Quito, Pichincha, Ecuador; 6 Centro de Investigación de la Biodiversidad y Cambio Climático, Universidad Tecnológica Indoamérica, Quito, Pichincha, Ecuador; 7 Saint Michael’s College, Colchester, VT, USA; 8 Tropical Herping, Quito, Pichincha, Ecuador; 9 Leibniz-Institut für Biodiversität der Tiere, Zoologisches Forschungsmuseum Alexander Koenig, Bonn, Germany

**Keywords:** New species, MicroCT scans, Phylogeny, Upper basin of the Pastaza river, Eastern Andean slopes

## Abstract

We describe a new species of terrestrial frog of the genus *Noblella* from the eastern versants of the Ecuadorian Andes in the upper Pastaza watershed. *Noblella naturetrekii* sp. n. differs from its Ecuadorian congeners by the presence of a differentiated tympanic membrane and a weakly defined tympanic annulus, and eyelids with rounded tubercles. In addition, the new species is characterized by its blackish–dark brown ventral coloration scattered with little white dots and the absence of papillae at the tip of the fingers and toes. We provide a detailed description of the call and osteology of the new species. Finally, we present the most complete phylogeny of the genus, which confirms that *Noblella* is a non-monophyletic group.

## Introduction

Knowledge of the diversity of amphibians in Ecuador is constantly increasing, with 609 species formally described to date (Ron, Merino-Viteri & Ortiz, 2019). On the other hand, deforestation, habitat change, climate change and mainly diseases (e.g., chytridiomycosis) threaten to cause the extinction of species before they are known to science (Gibbons et al., 2000; Todd, Willson & Gibbons, 2010; Scheele et al., 2019). Since 2006, the Ecominga Foundation has facilitated and conducted biodiversity research and conservation on the upper basin of the Pastaza River in Ecuador (Ríos-Alvear & Reyes-Puig, 2015; Reyes-Puig et al., 2019). The goal of the Foundation is to protect Andean ecosystems and function as an ecological corridor between Llanganates National Park and Sangay National Park, areas with high levels of biodiversity and endemism (Reyes-Puig et al., 2010, 2015, 2019; Salazar & Jost, 2012) that connect the eastern slopes of the Ecuadorian Andes with the Amazon Rainforest.

During the last 2 years, surveys in the upper basin of the Pastaza River have produced records of an unknown species of terrestrial frog of the genus *Noblella*, a taxon composed of 13 described species (Frost, 2019). *Noblella* is distributed in Colombia, Ecuador, Peru and Bolivia, mainly in the eastern and western Andean slopes, but also in the Amazonian basin (Frost, 2019). Currently, the genus faces a taxonomic uncertainty due to the lack of genetic information of the type series (Hedges, Duellman & Heinicke, 2008; De la Riva et al., 2017; Catenazzi & Ttito, 2018). Furthermore, it is likely some species of *Psychrophrynella* are nested within *Noblella* (Catenazzi & Ttito, 2019). Thus, the phylogenetic relationships of the genus *Noblella* and *Psychrophrynella* are still unknown. Herein, we formally describe a new species of terrestrial frog, which is found within the mosaic of Naturetrek Reserves of the Ecominga Foundation, on the eastern Andean slopes of central Ecuador. We support the validity of the new species with a set of morphological, acoustic, and molecular data. Finally, we present the most complete phylogeny to date of the genus *Noblella*.

## Materials and Methods

### Ethics statement

We conducted this research under collection permits N° 02-2018-IC-FAU-DPAT-VS, N° 01 2017-IC-FAU-DPAT-VS, N° 018-2017-IC-FAU-DNB/MAE, and agreement for access to genetic resources MAE-DNB-CM-2016-0045, issued by the Ministerio del Ambiente del Ecuador. We carried out this study in accordance with the guidelines for use of live amphibians and reptiles in field research (Beaupre et al., 2004), compiled by the American Society of Ichthyologists and Herpetologists, the Herpetologists’ League and the Society for the Study of Amphibians and Reptiles.

### Taxonomy and common name

We follow the family taxonomy proposed by Padial, Grant & Frost (2014). For recognizing species, we adopted the unified Species Concept (De Queiroz, 2005, 2007). The common name of the new species was formulated following the suggestions provided by Coloma & Guayasamin (2017).

The electronic version of this article in portable document format will represent a published work according to the International Commission on Zoological Nomenclature (ICZN), and hence the new names contained in the electronic version are effectively published under that Code from the electronic edition alone. This published work and the nomenclatural acts it contains have been registered in ZooBank, the online registration system for the ICZN. The ZooBank life science identifiers (LSIDs) can be resolved and the associated information viewed through any standard web browser by appending the LSID to the prefix http://zoobank.org/. The LSID for this publication is: LSID urn:lsid:zoobank.org:pub:3D663D4F-1AFF-4949-9C18-FFE37C97196A. The online version of this work is archived and available from the following digital repositories: PeerJ, PubMed Central, and CLOCKSS.

### Study area

**Figure 1:**
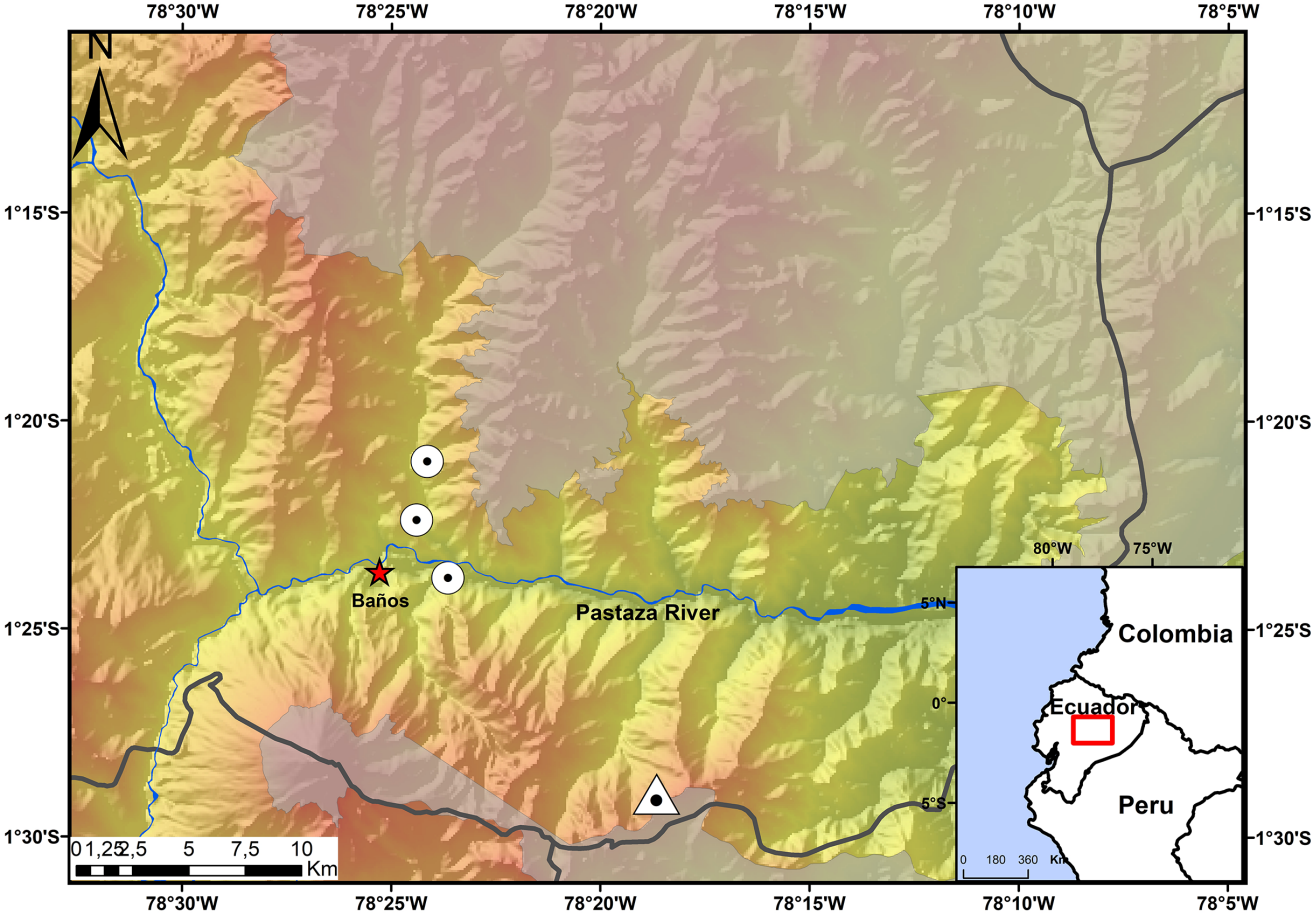
Map showing the four known localities of distribution of *Noblella naturetrekii* sp. n. The triangle symbolizes the type locality of *Noblella naturetrekii* sp. n. and the circles represent the localities of the paratypes of the new species. The gray line represents the provincial boundaries.

The Ecominga Foundation has established a system of reserves in the upper basin of the Pastaza River in the township of Baños, Tungurahua, Ecuador (Fig. 1). The foundation employs a group of community park rangers that actively participate in research and conservation of the reserves under the “Keepers of the Wild” program of the World Land Trust (UK). Specimens of the new species of *Noblella* were obtained by community park rangers in two of the protected areas by Ecominga Foundation: Vizcaya Naturetrek Reserve (1.39622°S, 78.39417°W; 2,400 m) and Bosque Protector Cerro Candelaria, Naturetrek Reserve Lote G (1.48272°S, 78.31096°W; 2,000 m); the remaining specimens were obtained outside the reserves, on a forest patch along the Baños-Vizcaya road (1.37305°S, 78.4067°W; 2,037 m). As a result of these initial observations, additional surveys were conducted at the Vizcaya Naturetrek Reserve, in collaboration with the Instituto Nacional de Biodiversidad of Ecuador (INABIO) and the study abroad School for International Training. Nocturnal and diurnal transects of 500 m were walked at the rate of 1 h/100 m. A 75 m line of plastic pitfall traps were also installed, using 10 twenty-gallon buckets placed at a depth of 60 cm every seven or eight m.

### DNA extraction, amplification and sequencing

We obtained DNA sequences of specimen DHCM 13307 to determine the phylogenetic relationships of the new species and other described *Noblella*. DNA was extracted from muscle or liver tissue preserved in 95% ethanol. Standard polymerase chain reaction (PCR) was performed to amplify DNA fragments for mitochondrial genes 12S rRNA, 16S rRNA and the nuclear genes recombination-activating 1 and Tyrosinase (TYR). PCR amplification was carried out following standardized protocols. Amplicons were sequenced in both directions by the Macrogen Sequencing Team (Macrogen Inc., Seoul, Korea). The sequences generated in this study are available at GenBank.

The new sequences were assembled and edited with Geneious 7.1.7 (GeneMatters Corp, Minneapolis, MN, USA). After assemblage, the new sequences were combined with sequences from GenBank for all *Noblella* and representative samples of all genera of Terrarana. We also added GenBank sequences of the nuclear gene TYR which are available for *Noblella* and other Terrarana. GenBank sequences were originally published by Faivovich et al. (2005), Lehr, Fritzsch & Mueller (2005), Heinicke, Duellman & Hedges (2007), Heinicke et al. (2009), Hedges, Duellman & Heinicke (2008), Padial, Castroviejo-Fisher & De la Riva (2009), Padial et al. (2012), Canedo & Haddad (2012), Motta et al. (2016), von May et al. (2017), and De la Riva et al. (2017). The final matrix, with 66 terminals and up to 3,751 bp, was imported to Mesquite version 2.75 (Maddison & Maddison, 2010). Sequences were aligned using the Muscle extension (Edgar, 2004) in Mesquite. The final alignment was inspected visually for errors that were adjusted manually.

### Phylogenetic analyses

Phylogenetic relationships were inferred for all genes concatenated using maximum likelihood as optimality criterion. Because different evolutionary processes have operated on each gene, we partitioned the matrix by gene and codon position to find the best model of evolution for each gene and codon position and then to find the optimal partition scheme. To accomplish both tasks, we used the command MFP+MERGE (Chernomor, von Haeseler & Minh, 2016; Kalyaanamoorthy et al., 2017) in software IQ-TREE multicore version 1.6.8 (Nguyen et al., 2015). To find the best phylogeny we ran 10 independent maximum likelihood searches using IQ-TREE 1.6.8 under default settings. To assess branch support, we made 200 non-parametric bootstrap searches also in IQ-TREE (-b command). *Eleutherodactylus coqui* was used to root the tree. Pairwise genetic distances between species (uncorrected-p) for gene 16S were calculated with MEGA 5. Compared sequences had at least 550 bp of overlap.

### Morphological data

Most specimens were euthanized with benzocaine, fixed in 10% formaldehyde and preserved in 75% ethanol. Before the preservation liver and leg muscle tissue samples were collected from all specimens. Two specimens (ZSFQ 933, 934) were fixed in 90% ethanol and preserved in 75% ethanol. The specimens were deposited in the herpetology collection of the Instituto Nacional de Biodiversidad (DHMECN) and the Museo de Zoología-COCIBA-USFQ (ZSFQ). Other species of *Noblella* were also examined (Appendix I).

All the collected specimens were measured in preservative using digital calipers to the nearest 0.01 mm, in the measurements the standard deviation follows the mean. To facilitate the comparisons with other species we utilized the sequence of characters proposed by Guayasamin & Terán-Valdez (2009) and we followed the proposal of Heinicke et al. (2018) for the systematic classification of family. These measurements include: snout to vent length (SVL, from the tip of the snout to the cloaca); head width; diameter of the eye, eye–nostril distance (from the anterior ocular angle to the posterior edge of the nostril); length of tympanum (smallest diameter), minimum interorbital distance, minimum eyelid width; hand length (from the posterior edge of the palmar tubercle to the tip of the third digit); ankle length; foot length (from the posterior edge of the external metatarsal tubercle to the tip of the fourth digit). Sexual maturity was determined by the presence of vocal slits or extended vocals sacs in males and by the presence of eggs or convoluted oviducts in females. We made an incision in order to determine the condition of the tympanum (Duellman & Lehr, 2009).

For the osteological description one paratype (DHMECN 14420) was scanned by use of a high-resolution micro-computed tomography (micro-CT) desktop device (Bruker SkyScan 1173, Kontenich, Belgium) at the Zoologisches Forschungsmuseum Alexander Koenig (ZFMK, Bonn, Germany). To avoid movements during scanning the specimen was placed in a small plastic container and mounted with styrofoam. The scan was conducted in 180 degrees at rotation steps of 0.3 degrees with a source voltage of 35 kV and a source current of 150 µA without the use of a filter at an image resolution of 21.3 µm. Scan duration was 19 min with an exposure time of 280 ms. The CT-dataset was reconstructed using N-Recon software (Bruker MicroCT, Kontich, Belgium) and rendered in three dimensions through the aid of CTVox for Windows 64 bits version 2.6 (Bruker MicroCT, Kontich, Belgium). Osteological terminology follows Duellman & Trueb (1994), Guayasamin & Terán-Valdez (2009), Trueb (1973), Scherz et al. (2017), and Suwannapoom et al. (2018). Cartilage structures were omitted from the osteological descriptions, because micro-CT does not render cartilage.

### Bioacoustics

Sound recordings were made by José Vieira with an Olympus LS-10 Linear PCM Field Recorder and a Sennheiser K6–ME 66 unidirectional microphone. The calls were recorded in WAV format with a sampling rate of 44.1 kHz/s with 16 bits/sample and analyzed with Raven Pro version 1.5 (Bioacoustics Research Program, 2017). All calls are stored at the Laboratorio de Biología Evolutiva at Universidad San Francisco de Quito (LBE). Measurements of acoustic variables were obtained as described in Köhler et al. (2017). A call is defined as the collection of acoustic signals emitted in sequence and produced in a single exhalation of air. A note is a temporally distinct segment within a call; notes are separated by a silent interval. Pulsed notes are those having one or more clear amplitude peaks while tonal notes have relatively constant amplitude throughout the call; in a call, pulses are not separated by a fully silent interval. A call series (or call group) is defined as a sequence of calls that is separated from other such groups by periods of silence much longer than the inter-call intervals, which are stable or changing in a predictable pattern (see Köhler et al., 2017).

## Results

**Figure 2:**
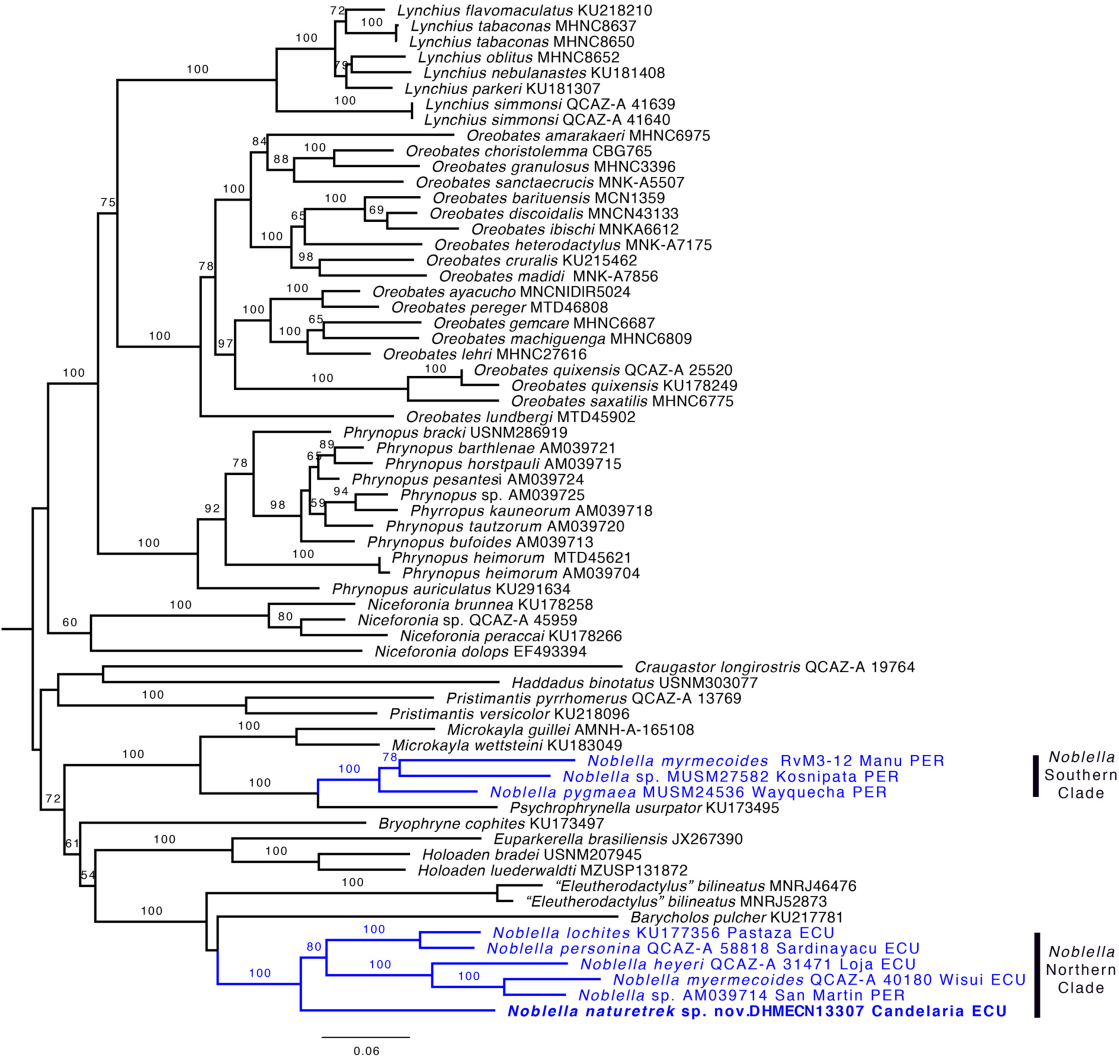
Phylogeny of *Noblella* (blue) showing the relationships of *Noblella naturetrekii* sp. n. Phylogeny of *Noblella* (blue) showing the relationships of *Noblella naturetrekii* sp. n. The phylogeny was based on 3,751 bp of mitochondrial (gene fragments 12S and 16S) and nuclear (gene fragments RAG1 and Tyrosinase) DNA sequences. Branch support is presented as non-parametric bootstrap (values <50 are not shown). For each individual, museum catalog number or, if unavailable, GenBank accession number is shown. GenBank accession numbers consist of two letters followed by six digits except for numbers beginning with “KU” which correspond to specimens from the University of Kansas Natural History collection. The outgroup is not shown.

**Phylogenetic relationships and genetic distances.** The genus *Noblella* was inferred as polyphyletic (Fig. 2) and is formed by two distinct clades. One clade is closely related to *Psychrophrynella* and *Mycrokayla*, which includes *Noblella myrmecoides*, *N. pygmaea*, and *Noblella* sp. This clade is distributed in southern Peru. The other clade is closely related to *Barycholos* and “*Eleutherodactylus*” *bilineatus* and includes *N. heyeri*, *N. lochites*, *N. myrmecoides, N. naturetrekii* sp. n., *N. personina*, and *Noblella* sp. from San Martín, Peru. *N. myrmecoides* is present in both clades, highlighting the need for more exhaustive sampling with the inclusion of topotipic material. One of the terminals can represent an undescribed species related with *N. myrmecoides*. Samples from this clade occur in Ecuador and northern Peru. Support for the paraphyly of *Noblella* is strong because the bootstrap value for the node *Noblella* + *Microkayla* (Southern Clade) is 99 and for the node *Noblella* + *Barycholos*- “*Eleutherodactylus*” (Northern Clade) is 100.

The new species is sister to all remaining species of the Northern Clade (Fig. 2). Uncorrected *p* genetic distances between *N. naturetrekii* sp. n. and other species of the Northern Clade range from 9% to 14% (gene 16S). Its phylogenetic position, high genetic distances, and morphological distinctiveness (see Diagnosis) demonstrate that *N. naturetrekii* sp. n. is, in fact, a new species that we describe in the following section.

## Systematics accounts

**Figure 3:**
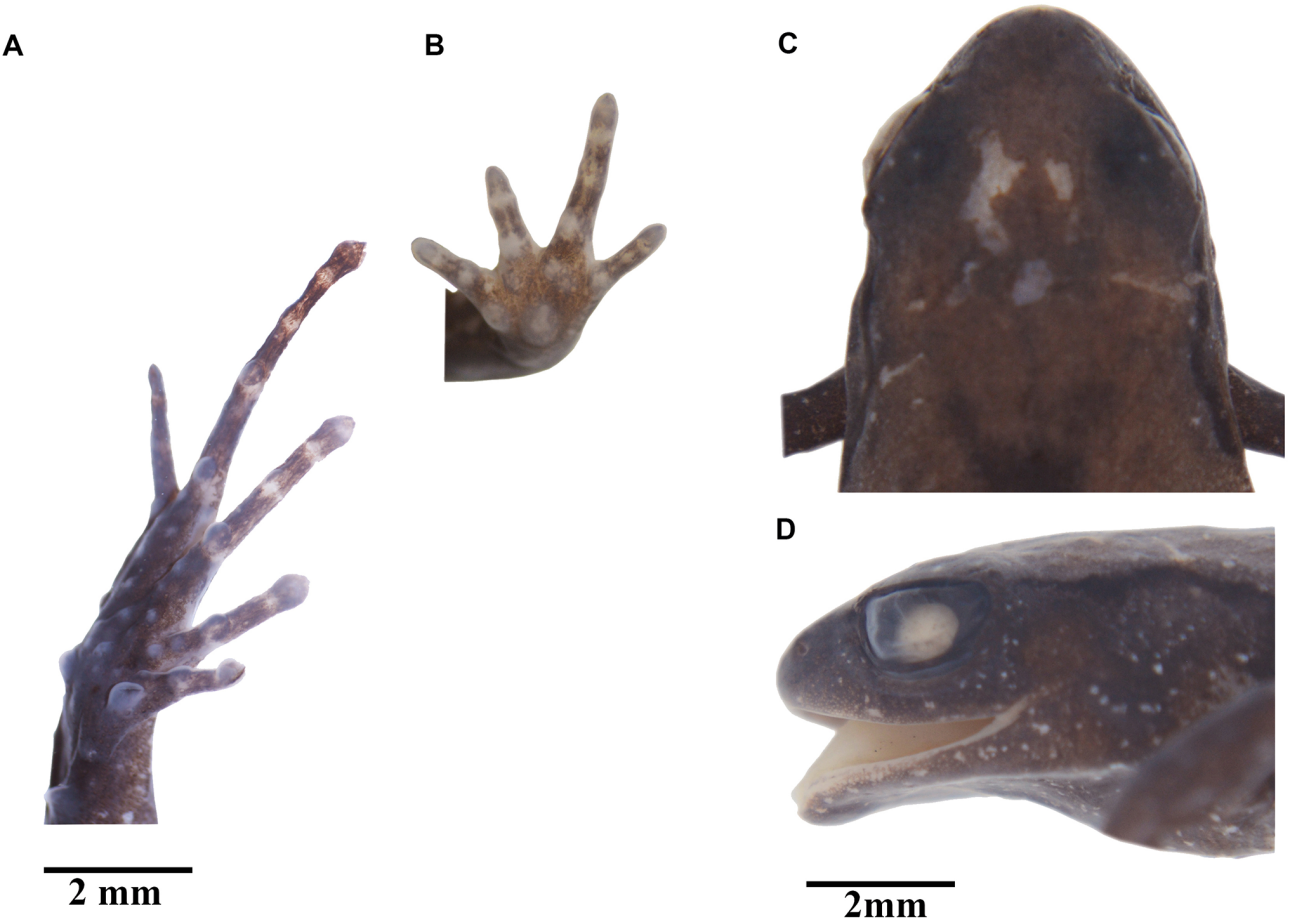
*Noblella naturetrekii* sp. n. Preserved holotype, DHMECN 13390, adult female, SVL = 14.2 mm; and paratype ZSFQ 934, adult female, SVL = 14.1 mm. (A) Palmar (holotype) surface; (B) plantar (paratype) surface; (C) dorsal view of the head (holotype); (D) lateral view of the head (holotype). Photographs by Carolina Reyes-Puig (A), and Juan Pablo Reyes-Puig (B–D).

**Figure 4:**
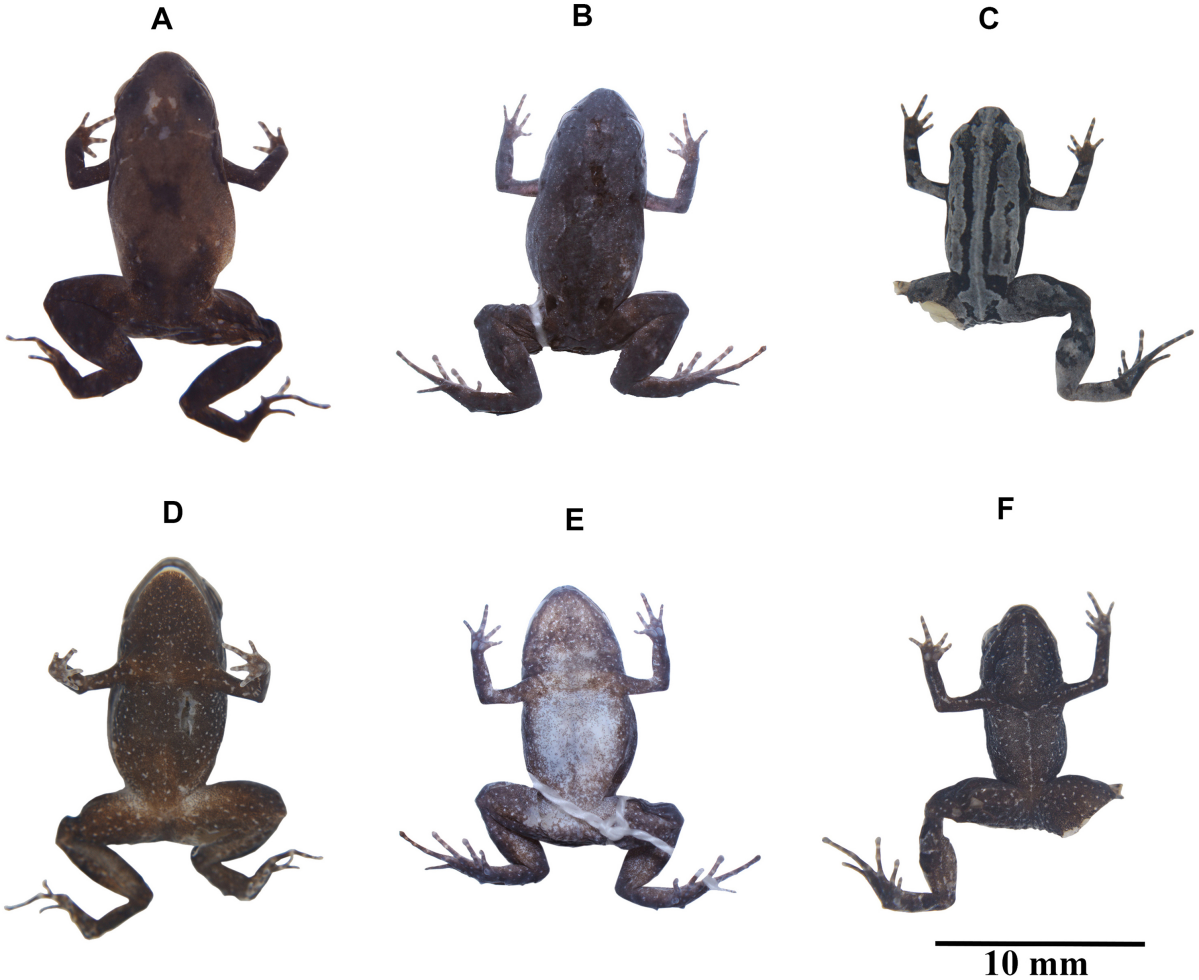
Variation in coloration of preserved *Noblella naturetrekii* sp. n. (A) and (D) DHMECN 13390, holotype, adult female, SVL = 14.2 mm; (B) and (E) ZSFQ 934, paratype, adult female, SVL = 14.1 mm; (C) and (F) DHMECN 14420, paratype, adult female, SVL = 13.1 mm. Photographs by Juan Pablo Reyes-Puig (A), (C) and (D), (F), and Carolina Reyes-Puig (B) and (E).

**Figure 5:**
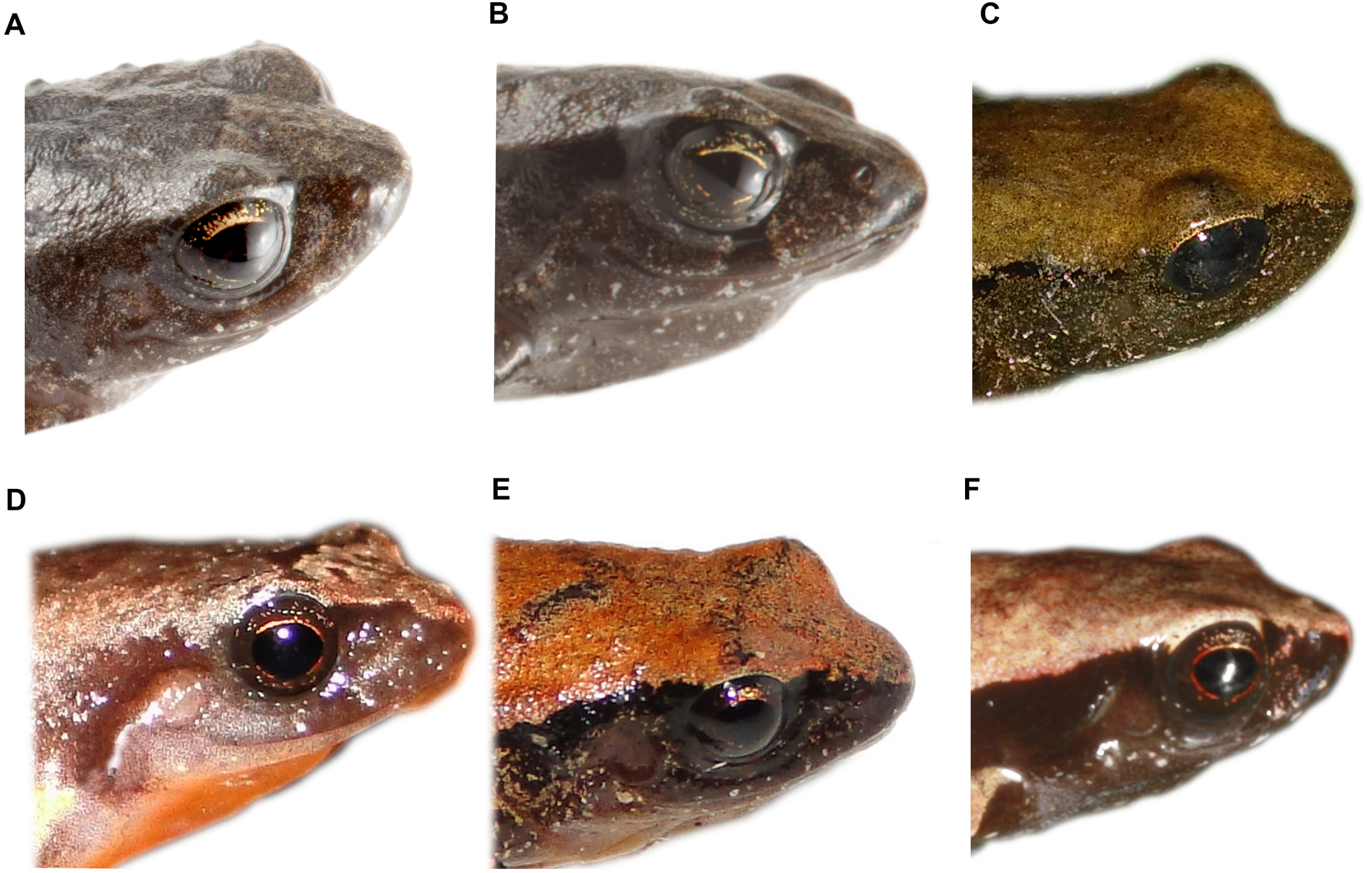
Detail of the tympanic annulus and tympanic membrane of *Noblella* from the eastern slopes of Ecuador. (A) *Noblella naturetrekii* sp. n, paratype, female, ZSFQ 934; (B) *N. naturetrekii* sp. n, paratype, male, ZSFQ 933; (C) *N. naturetrekii* sp. n, holotype, female, DHMECN 13390; (D) *N. personina*, female, EPN 14329 (Harvey et al., 2013); (E) *N. lochites*, female, EPN 14254 (Harvey et al., 2013); (F) *N. myrmecoides* from Jenaro Herrera, Loreto. Photographs by José Vieira (A) and (B), Juan Pablo Reyes-Puig (C), Jorge Brito M. (D) and (E) and Mauricio Ortega (F).

**Figure 6:**
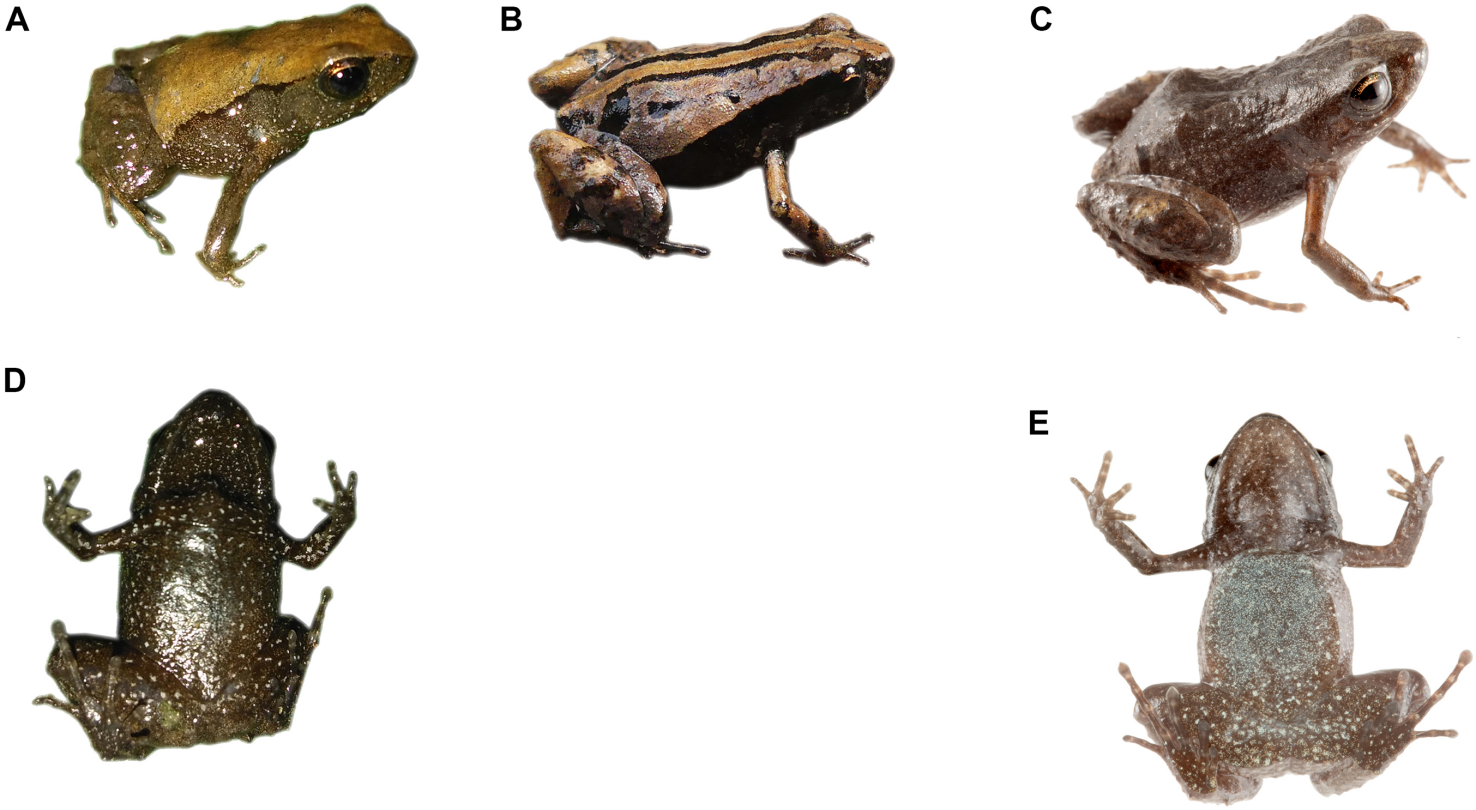
Dorsal and ventral patterns of *Noblella naturetrekii* sp. n in life. (A) and (D) Dorsal pattern and ventral pattern of DHMECN 13390, holotype, adult female, SVL = 14.2 mm; (B) Dorsal pattern of DHMECN 14420, paratype, adult female, SVL = 13.1 mm; (C) and (E) Dorsal pattern of ZSFQ 934, paratype, adult female, SVL = 14.1 mm. Photographs by Juan Pablo Reyes-Puig (A) (B) and (D) and José Vieira (C) and (E).

**Figure 7:**
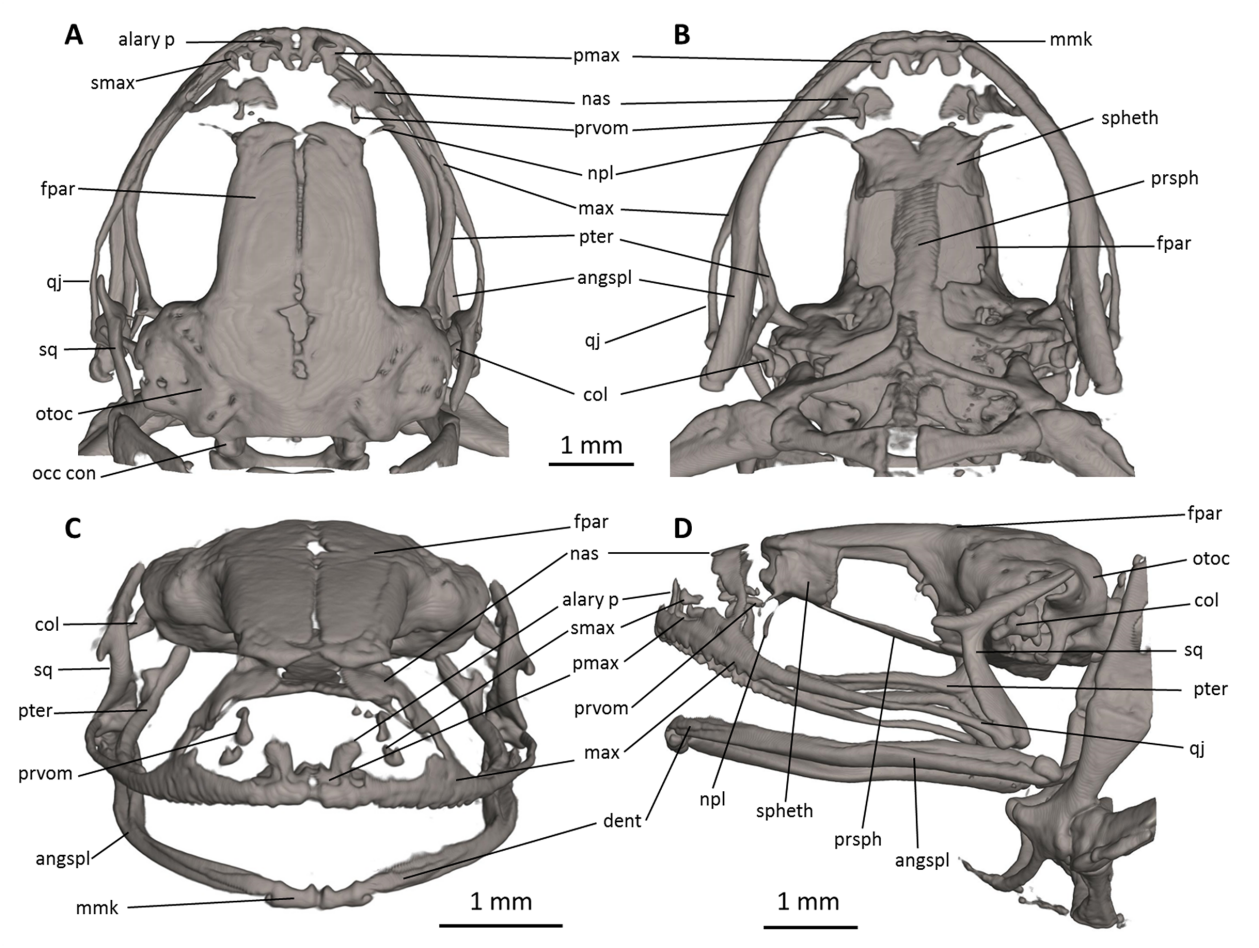
Head skeleton of *Noblella naturetrekii* sp. n. (paratype, DHMECN 14420). The skull is shown in (A) dorsal, (B) ventral, (C) frontal, and (D) lateral views. alary p, alary process; angspl, angulosplenial; col, columella; dent, dentary; fpar, frontoparietal; max, maxilla; mmk, mentomeckelian bone; nas, nasal; npl, neopalatine; occ con, occipital condyle; otoc, otoccipital (fused prootic and exoccipital); pmax, premaxilla; prsph, parasphenoid; prvom, prevomer; pter, pterygoid; qj, quadratojugal; smax, septomaxilla; spheth, sphenethmoid; sq, squamosal.

**Figure 8:**
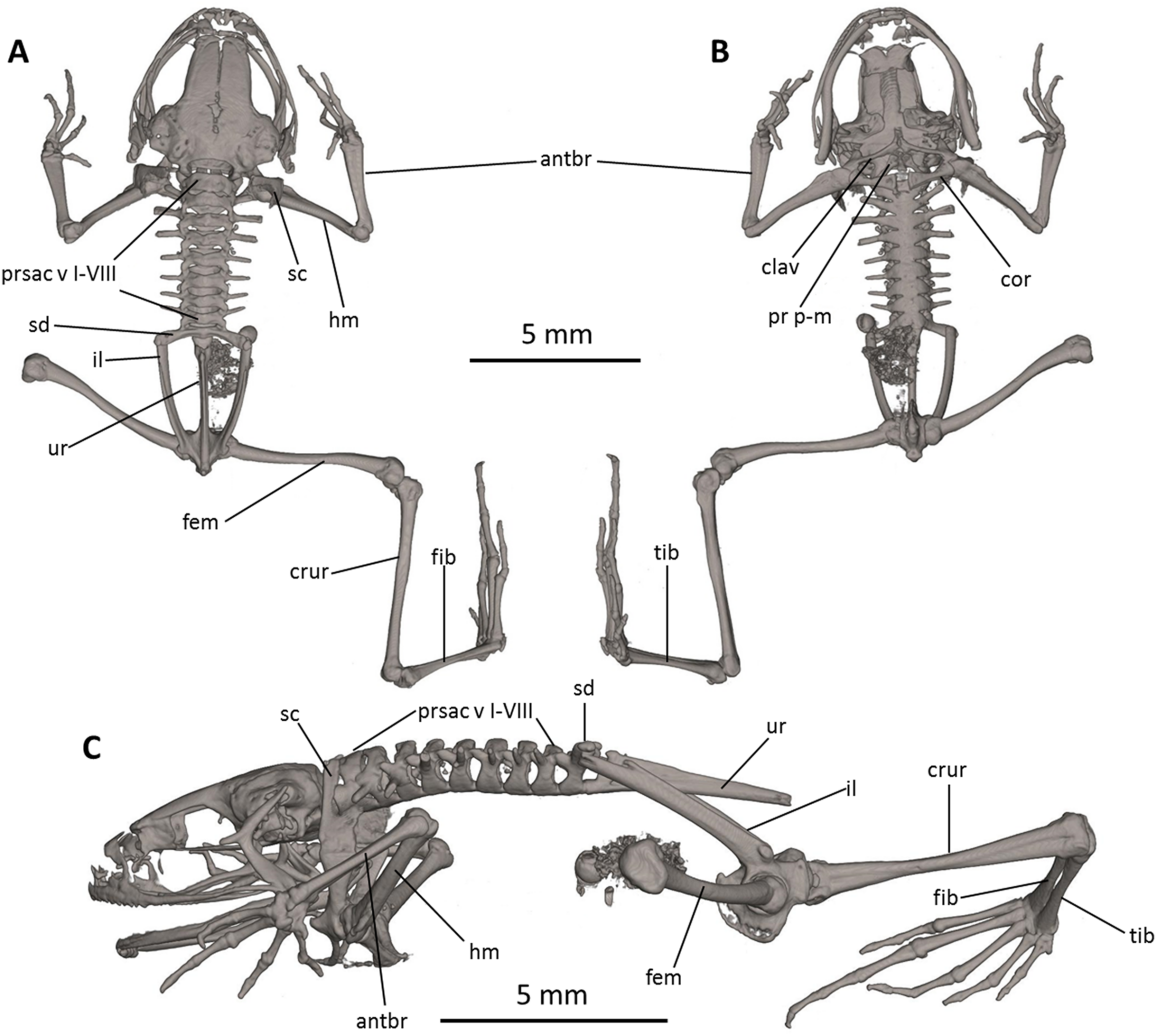
Osteology of *Noblella naturetrekii* sp. n. (paratype, DHMECN 14420). The full skeleton is shown in (A) dorsal, (B) ventral, and (C) lateral views. antbr, os antebrachii (radius + ulna); clav, clavicle; cor, coracoid bone; crur, os cruris (tibia + fibula); fem, femoral bone; fib, fibulare; hm, humeral bone; il, ilium; pr p-m, processus postero-medialis; prsac v, presacral vertebrae I-VIII; sd, sacral diapophysis; sc, scapula; ur, urostyle; tib, tibiale.

**Figure 9:**
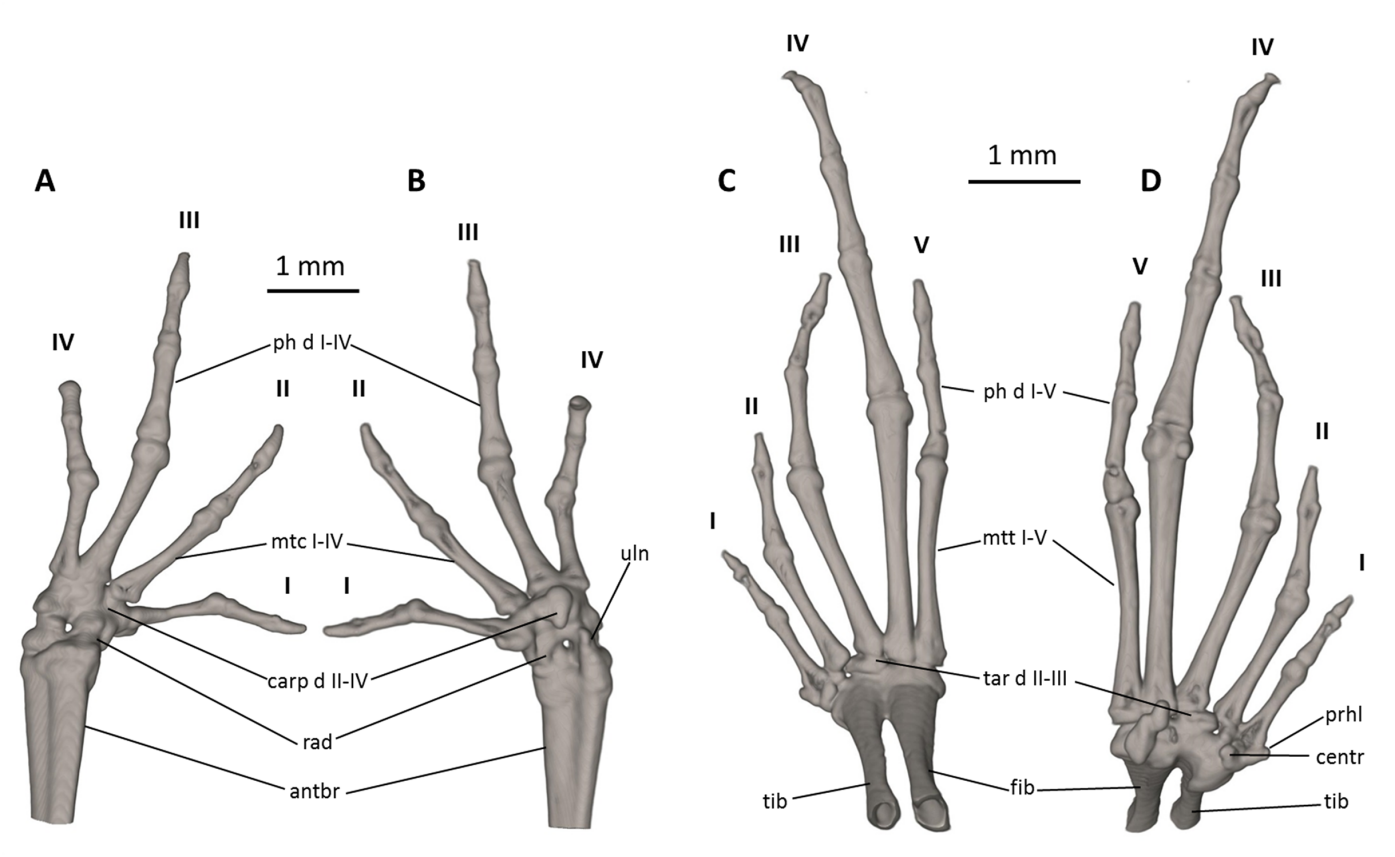
Osteology of the limbs of *Noblella naturetrekii* sp. n. (paratype, DHMECN 14420). The left forelimb is shown in (A) dorsal, and (B) palmar aspects; and the right foot in (C) dorsal, and (D) plantar aspects. Digits numbered I–V. antbr, os antebrachii (radius + ulna); carp d II–IV, carpale distale F2–F4; cent, centrale; fib, fibulare; mtc I–IV, metacarpalia F1–F4; mtt I–V, metatarsalia T1–T5; ph d I–IV, finger phalanges F1–F4; ph d I–V, toe phalanges F1–F5; prhl, prehallux, rad, radiale; tar d II–III, tarsale distale T2–T3; tib, tibiale; uln, ulnare.

***Noblella naturetrekii* new species.**
Figs. 3–9

LSID urn:lsid:zoobank.org:act:64223F8F-6FF2-4A45-B63D-1F0CE22C1113

**Proposed standard English name.** Naturetrek Leaf Frog
**Proposed standard Spanish name.** Cutín Noble de Naturetrek

**Holotype.** DHMECN 13390 (Figs. 3 and 4), adult female, collected in Bosque Protector Cerro Candelaria (1.428722°S, −78.30421°W; 2,000 m, Fig. 1), Naturetrek Reserve, Río Verde, Cantón Baños, Tungurahua Province, by JPRP, Mindee Goodrum, and Jordy Salazar on April 24, 2017.

**Paratypes** (five females, three males). DHMECN 13391 same data as the holotype; DHMECN 13307 female, collected in the Naturetrek Reserve Vizcaya, Ulba, Cantón Baños, Tungurahua province, Ecuador (1.3962203°S, 78.3941767°W; 2,400 m), by JPRP and Fausto Recalde on September 12, 2016; DHMECN 14493 female, DHMECN 14494 male, collected in the same location as DHMECN 13307, by JPRP and Stalin Peña on March 10, 2018; DHMECN 14420, female, collected in the same location as DHMECN 14493, by JPRP and Stalin Peña on July 13, 2018. DHMECN 14411, male, Naturetrek Reserve Vizcaya, Ulba, Baños, Tungurahua, Ecuador (1.349582°S, 78.402425°W; 2,400 m), by MYM, JPRP and Daniela Franco, collected on March 14, 2018; DHMECN 14420 female, same data as previous; ZSFQ 933 male and ZSFQ 934 female, in a forest patch on the Baños-Vizcaya road (1.37305°S, 78.4067°W; 2,037 m), Tungurahua province, Ecuador by JV, Claudia Herrera, Josue Picho, and Jorge Castillo on July 14, 2018.

**Generic placement.** A species of terrestrial frog of the genus *Noblella* as defined by Hedges, Duellman & Heinicke (2008): head no wider than body; cranial crests absent; tympanic membrane differentiated (except in *N. duellmani*); dentigerous processes of vomers absent; terminal discs on digits not or barely expanded; discs and circumferential grooves present distally; terminal phalanges narrowly T-shaped; Finger I shorter than, or equal in length to, Finger II; Finger IV containing only two phalanges in *N. carrascoicola*, *N. lochites*, *N. myrmecoides*, *N. ritarasquinae*, and in *N. naturetrekii*. Toe III shorter than Toe V; tips of at least Toes III–IV acuminate; subarticular tubercles not protruding; dorsum pustulate or shagreen; venter smooth; SVL less than 22 mm. *N. pygmaea* (SVL 11.1–12.4 mm in adults) (Lehr & Catenazzi, 2009) and *N. naturetrekii* (SVL 12.1–14.2 mm in adults) have the smallest SVL of the genus. Nevertheless, it is important to mention that there are no known synapomorphies for *Noblella* or for *Psychrophrynella*. These two genera are morphologically similar and are closely related (De la Riva et al., 2017; Catenazzi & Ttito, 2018).

**Diagnosis.** The new species differs from its congeners by the combination of the following characteristics: (1) skin of the dorsum and flanks shagreen to tubercular (smooth by preservation effects), with scattered subconical tubercles on the sacral region; finely granular skin on the abdomen; (2) tympanic membrane differentiated; tympanic annulus present, weakly defined (Fig. 5); (3) snout elongated in dorsal view, rounded in lateral view; (4) eyelids with flattened and rounded tubercles; (5) dentigerous processes of the vomers absent; (6) vocal slits and sac present, nuptial pads present; (7) fingers not expanded distally, finger tips acuminate but lacking papillae; Finger I smaller than Finger II, without circumferential grooves and without papillae (Fig. 3); (8) distal phalanges blunt or T-shaped, phalangeal formula of hand 2, 2, 3, 2; (9) supernumerary palmar tubercles absent, ulnar tubercles present (reduced by preservation effects); (10) one subconical tarsal tubercle present (Fig. 3); two prominent metatarsal tubercles; toe tips pointed, with slightly defined circumferential grooves and lacking papillae; (11) Toe V shorter than Toe III; (12) phalangeal formula of feet: 2, 2, 3, 4, 3 (Fig. 9); (13) in ethanol, dorsum brown with dark oblique lateral line that delineates flanks, large suprainguinal marks, dark brown to black scattered with numerous small white dots; in life dorsum predominately light or dark brown with two black suprainguinal marks or a mid-dorsal cream-colored stripe, the venter and throat is light brown to brown interspersed with small white spots; (14) SVL in males 11.2–12.1 (*n* = 3); and females 13.1–14.4 (*n* = 6).

**Comparisons.** *Noblella naturetrekii* differs from its Ecuadorian congeners by the presence of a differentiated tympanic membrane and tympanic annulus weakly defined, absence of papillae, presence of rounded and flattened tubercles on the eyelid, grayish-brown ventral coloration splattered with white spots and by having dorsal skin shagreen to tubercular in life. All currently described species of *Noblella* from Ecuador do not possess tubercles on the eyelids, and also have a visible tympanic annulus and tympanic membrane (Fig. 5). *N. naturetek* could be confused with other similar species that possess suprainguinal spots and a dark mask that extends to the inguinal region, such as the case of *N. coloma* (Guayasamin & Terán-Valdez, 2009) and *N. heyeri* (Lynch, 1986); however, those species exhibit visible and distinctive tympanic membrane and annulus. Moreover, *N. coloma* has a striking reddish ventral coloration (brown to blackish in *N. naturetrekii*). All the previously described species distributed on the eastern slope of the Andes, *N. myrmecoides* (Lynch, 1976), *N. personina* (Harvey et al., 2013), *N. lochites* (Lynch, 1976), have light ventral colorations and lack tubercles on the dorsum and eyelid, *N. myrmecoides* also possesses papillae at the tip of the digits (absent in *N. naturetrekii*) and the males of *N. personina* do not have vocal slits (present on *N. naturetrekii*). *N. duellmani* lacks a tympanic annulus and tympanic membrane (Lehr, Aguilar & Lundberg, 2004) (tympanic membrane differentiated and tympanic annulus weakly defined in *N. naturetrekii*), also, this species has three phalanges in the fourth finger (two in *N. naturetrekii*) and the ventral surfaces of shanks and thighs are brown with brownish orange spots (dark brown to black scattered with numerous small white dots in *N. naturetrekii*).

*Noblella naturetrekii* differs from *N. lynchi* (Duellman, 1991), *N. madreselva* (Catenazzi, Uscapi & von May, 2015), *N. peruviana* (Noble, 1921), *N. pygmaea* (Lehr & Catenazzi, 2009), and *N. thiuni* (Catenazzi & Ttito, 2019) by having two phalanges on Finger IV. *N. ritarasquinae* (Köhler, 2000) is characterized by having terminal papillae (absent in *N. naturetrekii*) and *N. carrascoicola* (De la Riva & Köhler, 1998) by having dorsum smooth (shagreen to tubercular in *N. naturetrekii*). Dorsally *Psychrophrynella chirihampatu* (Catenazzi & Ttito, 2016) and *Psychrophrynella glauca* (Catenazzi & Ttito, 2018) can be similar to *N. naturetrekii*, nevertheless, ventral surfaces of the new species are dark brown interspersed with small white spots (yellow in *Psychrophrynella chirihampatu* and red or reddish-brown in *Psychrophrynella glauca*).

**Description of the Holotype.** Female (DHMECN 13390). Head as long as wide, snout elongated in dorsal view and rounded in lateral view; canthus rostralis slightly concave in dorsal and lateral view; flat loreal region; upper eyelid is 69% of the interorbital distance, with two to three rounded and flattened tubercles over the eyelid; eye-nostril distance is 53% of the diameter of the eye; tympanic membrane differentiated and tympanic annulus weakly defined, supratympanic fold absent. Dentigerous processes of vomers absent. Dorsal skin shagreen (smooth by preservation effects) with small tubercles under the sacral region, ventral skin finely granular; ulnar tubercles absent; palmar tubercles rounded, double the size of the thenar tubercle; a rounded subarticular tubercle in each of the bases of the fingers I-II-III and IV; inconspicuous subarticular tubercles, slightly risen and rounded, fingers not extended distally, finger tips pointed and lacking papillae, circumarginal grooves absent; relative length of the fingers I < IV < II < III. A subconical tubercle prominent on the ventral surface of the tarsus, row of three low tubercles on the external posterior surface of the tarsus; two metatarsal tubercles, low and rounded; subarticular tubercles prominently rounded, without supernumerary plantar tubercles. Digits of the feet slightly expanded and acuminate, without papillae; distal portion of toe IV with poorly defined circumarginal groove; relative length of the toes I < II < III < IV > V. The measurements of the type series are listed in Table 1.

**Table 1:** Measurements (in mm) of type series of *Noblella naturetrekii* sp. n. Ranges followed by mean and standard deviation in parentheses.

Characters	Females (*n* = 6)	Males (*n* = 3)
SVL	13.1–14.4 (13.7 ± 0.5)	11.2–12.1 (11.6 ± 0.4)
HL	4.2–6.1 (4.7 ± 0.8)	3.9–4.2 (4.1 ± 0.1)
HW	4.2–5.4 (4.7 ± 0.4)	3.7–4.1 (3.9 ± 0.2)
ED	1.6–2.0 (1.8 ± 0.2)	1.1–1.6 (1.4 ± 0.2)
EN	0.9–1.0 (1 ± 0.1)	0.8–1.1 (0.9 ± 0.1)
MWE	1.0–1.5 (1.2 ± 0.2)	0.7–1.0 (0.8 ± 0.2)
TD	0.5–0.8 (0.64 ± 0.2)	0.5–0.7 (0.66 ± 0.1)
MIOD	1.3–2.5 (1.7 ± 0.5)	1.1–2.2 (1.5 ± 0.5)
LH	2.5–2.8 (2.6 ± 0.1)	2.3–2.4 (2.4 ± 0.1)
LS	3.8–6.5 (4.9 ± 1.2)	3.2–5.8 (4.1 ± 1.3)
LF	5.2–5.9 (5.6 ± 0.3)	4.9–5.1 (5.0 ± 0.1)

**Coloration of the holotype in alcohol (Fig. 4).** Dorsum light brown with tonalities of dark brown diffuse on the eyelids, mid back and two suprainguinal marks more defined; extremities dark brown; canthal region and flanks, dark brown interspersed with small white spots that extend obliquely from the posterior region of the tympanum to the insertion of the posterior limb; throat, belly, and other ventral surfaces dark brown interspersed with small white spots.

**Coloration in life of the holotype (Fig. 6).** Dorsum yellowish brown with diffuse grayish brown spots in the mid dorsum and posteriorly, two dark brown suprainguinal spots; a thin and irregular black line delineates the loreal region, extending obliquely to the insertion of the posterior limb; the flanks are dark brown interspersed with small white spots. Throat and all ventral surfaces are dark brown interspersed with small white spots.

**Variation of color patterns and external morphology (Figs. 4 and 6).** Based on our collection efforts we can recognize currently two dorsal coloration patterns in *N. naturetrekii*. The first consists of a dorsum predominately light or dark brown in faded tones with two black suprainguinal marks. The second pattern consists of a light brown dorsum with a mid-dorsal cream-colored stripe that extends from the point of the snout to the cloaca, delineated by two paravertebral dark brown to black stripes extending from the eyelid to join with the suprainguinal marks. This striped morph tends to possess a ventral cross pattern formed by the alignment of the small white spots through the superior extremities and the midline. The DHMECN specimens have a shagreen dorsum in life, while the ZSFQ specimens have a tuberculate dorsum. The male ZSFQ 933 has nuptial pads slightly defined, light brown, present in the external border of Finger I. The female ZSFQ 934 has the lower portion of the tympanic annulus slightly visible. It is important to mention that the preservation can affect the morphological characters, mainly the condition of the tympanum. Several specimens (DHMECN) have reduced the visibility of the tympanic annulus (which is weakly defined in life). This can be a result of the specimen manipulation or by the size of individuals.

**Osteology.** The osteological description is based on micro-CT images of an adult female paratype (DHMECN 14220). Details of the skull morphology are presented in Fig. 7, main skeletal features are shown in Fig. 8 and osteological aspects of the hand and foot are shown in Fig. 9.

**Skull (Fig. 7).** The skull is slightly longer than wide; the widest part is at the quadratojugal at about the anterior tip of the squamosal and is 97% the length of the skull. The rostrum is short; the distance from the anterior edge of the frontoparietals to the anterior face of the premaxilla is 23% of the skull length. At the level of midorbit, the braincase is about 39% of the maximum skull width.

The braincase combines well- and poorly-ossified elements. The prootic and exoccipital are fused to form the otoccipital. The frontoparietals are well-developed bones, distinctly longer than broad, slightly narrower anteriorly than posteriorly; they are separated along the anterior half of their length; there is a non-ossified fontanelle between the posterior half of the frontoparietals. The posterior portion of the braincase is fully enclosed, by the complete fusion of the frontoparietals with the otoccipitals. Anteriorly, the frontoparietals are in contact with the sphenethmoid. The sphenethmoid is well-ossified and ventrally fused at midline. Its posterior margin does not reach the midpoint of the orbit and it is broadly separated from the otoccipitals and is in ventral contact with the parasphenoid. The otoccipitals are ventrally fused with the parasphenoid alae. The cultriform process of the parasphenoid is about 31% the width of the braincase at mid-orbit. The lateral margins of the process are approximately parallel. The parasphenoid alae are long (each one distinctly longer than the cultriform process is wide at mid-orbit). The neopalatines are very thin. They articulate with the sphenethmoid but are not in contact with the maxilla. The septomaxilla is small. The columella (or stapes) is large and well ossified.

The dorsal investing bones are poorly developed. The nasals are thin and broadly separated from one another; they are posteriorly broadly separated from the anterior end of the frontoparietals and laterally in thin contact with the maxilla. The small prevomers are broadly separated from one another medially.

The maxillary arcade bears many small, poorly resolved teeth on the premaxillae and maxillae. The premaxilla is partially separated medially, and their anterodorsal alary processes rise weakly divergent from the midline, but are still distinctly separated from the nasals. The premaxilla and maxilla are in lateral contact via a simple, juxtaposed articulation. The posterior end of the maxilla is acuminate and in contact with the quadratojugals. The triradiate pterygoid bears a long, curved anterior ramus that is oriented anterolaterally toward the maxilla, with which it articulates at the anteroventral corner of the orbit. The posterior ramus of the pterygoid is slightly longer than the medial ramus; however, the latter is slightly more robust than the posterior ramus. The edge of the medial ramus overlaps the lateral edge of the otoccipital. The quadratojugal is slender and articulates with the ventral ramus of the squamosal. The squamosal is T-shaped; the otic ramus is much longer than the zygomatic ramus. The mandible is slim and edentate. The mentomeckelians are small, medially and laterally slightly broadened, and medially contact with another by strips of cartilage. The angulosplenial is long and arcuate and articulates broadly with the relatively small and thin dentaries. The coronoid process is a relatively long and strongly raised ridge. The dentary is posteriorly acuminate and contacts the mentomeckelian bones. The only ossified portions of the hyoid apparatus are the two posteromedial processes, which are moderately expanded anteriorly and slightly expanded posteriorly and are moderately separated from one another at the anterior ends.

**Postcranium (Fig. 8).** There are eight presacral vertebrae. All of the presacrals are non-imbricate. First presacral vertebra is longer than posterior vertebrae. All except presacral I bear well developed diapophyses. The transverse processes of presacral V–VIII are similar in size, with those of Presacral III being the longest, those of presacral IV being the second longest, and those of Presacral II being the shortest is relatively wider than the other presacral vertebrae. The transverse processes of Presacral II, VII, and VIII have a slightly anterolateral orientation. The transverse processes of presacrals II–IV are thicker and broader than those of presacrals V–VIII. The sacrum with only very slightly expanded diapophyses. The urostyle is long, slender, slightly shorter than the presacral portion of the vertebral column and bears a well-pronounced dorsal crest. The bone has a bicondylar articulation with the sacrum.

In the pectoral girdle, the clavicles are long and slim, oriented anteromedially, not curved, with the medial tips narrowly separated from one another. The coracoids are stout, with the anterior edge curved, the medial tips of the coracoids are broadly separated from another. The glenoidal and sternal ends of the coracoid are about equally expanded. The scapula is long with a prominent pars acromialis that is not separated from the pars glenoidalis. The cleithrum is not visible in the micro-CT scans. The sternum has no ossified elements. Omosternum absent.

In the pelvic girdle, the long, slender ilial shafts bear conspicuous dorsolateral crests along most of their length.

**Manus and pes (Fig. 9).** All phalanges are ossified with a phalangeal formula for the fingers and toes: 2-2-3-2 and 2-2-3-4-3 (both standard), respectively. The increasing order of finger length: I < IV < II < III, and that of toes is: I < II < V < III < IV. Small distal knobs are present on terminal phalanges of fingers III and IV and toes III and IV, and maybe others, but they are not always well resolved in the micro-CT scans and are sensitive to the thresholds used during reconstruction. Terminal phalanges of all toes and fingers narrower than penultimate phalanges of all toes and fingers, respectively. Carpus and tarsus are not well resolved in the micro-CT scans, and it is difficult to distinguish the different elements of their composition.

**Figure 10:**
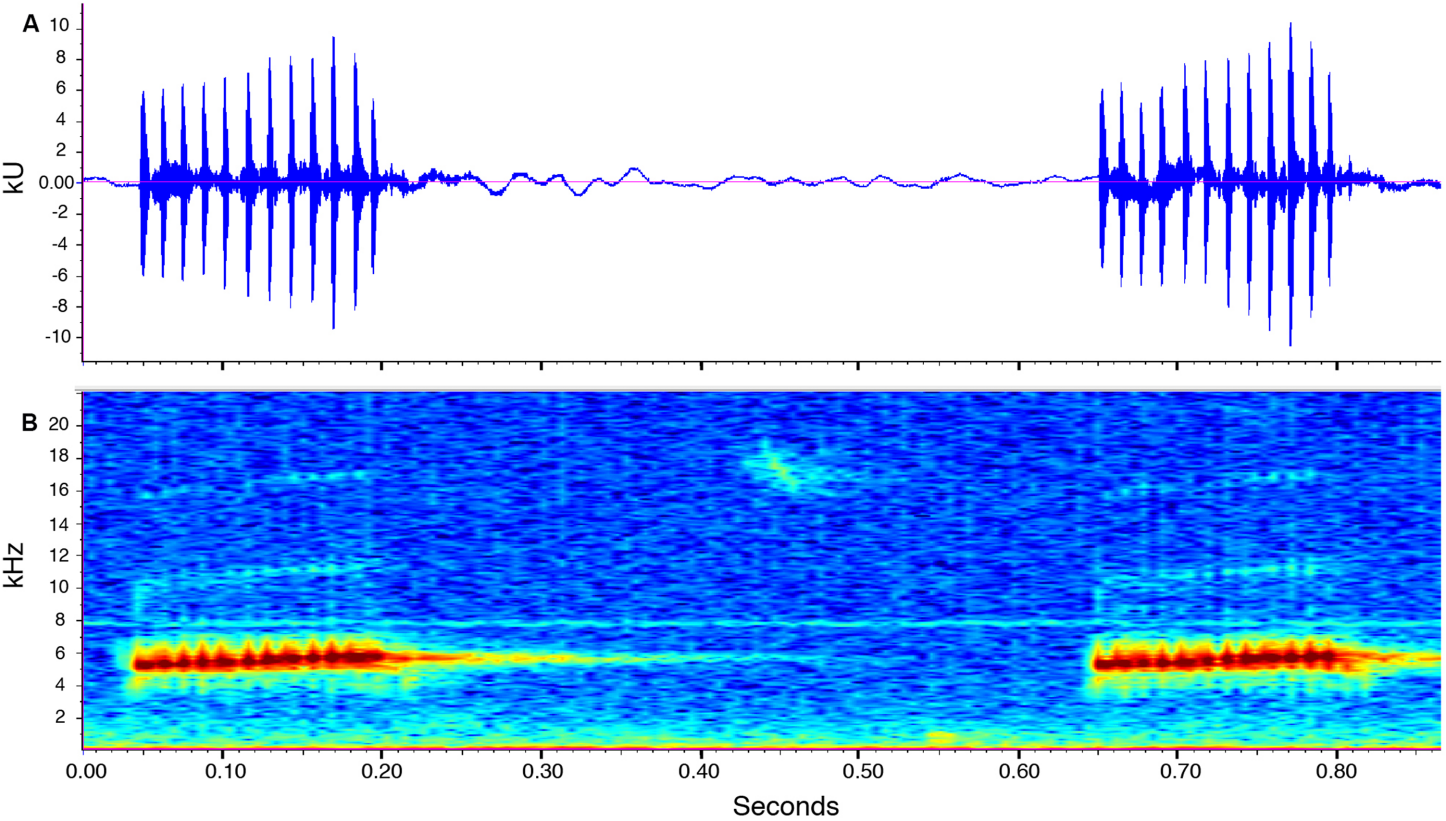
Call of the paratype (ZSFQ 933) of *Noblella naturetrekii* sp. n. The recording was made at 21:40 pm on July 14, 2018 (air temperature not recorded). (A) Amplitude; (B) Frequency.

**Call description (Fig. 10).** Call recorded by José Vieira, the recording was made at 21:40 pm on July 14th, 2018 (air temperature not recorded). Adult male ZSFQ 933 (recording code: LBE-C-048). Calls are produced in series. Each series contains four to 34 calls (mean = 12.2 ± 11.5; *n* = 6). Time between call series is highly variable, from 6.7 to 21.8 s (mean = 13.9 ± 8.2; *n* = 4). The call sounds like running a thumb over a comb. Each call has a duration of 0.1–0.2 s (mean = 0.1 ± 0.01; *n* = 16) and is markedly pulsed (10–13 pulses per call, mean = 11.6 ± 0.8; *n* = 16). Each call is frequency modulated, with its dominant frequency slightly increasing through time. The dominant frequency at the beginning of each call is at 4,980–5,353 Hz (mean = 5,175.6 ± 100.2; *n* = 16) and at 5,661–5,771 Hz (mean = 5,723.3 ± 30.7; *n* = 16) at the end. In most calls, harmonics are visible; the first harmonic is at 9,865–11,475 Hz (*n* = 14), and the second harmonic is at 15,390–17,157 Hz (*n* = 13).

**Distribution and Natural History.** The new species, *N. naturetrekii* is known from four localities of cloud forest in the upper Pastaza watershed in the Llanganates-Sangay Ecological Corridor (Fig. 1). Three localities are part of the Naturetrek Reserves of the Ecominga Foundation, in Vizcaya and in the Bosque Protector Cerro Candelaria, which are found in the eastern region of the Baños township, Tungurahua Province. *N. naturetrekii* thus makes itself a symbol of conservation of the ecological corridor between Llanganates National Park and Sangay National Park. The new species is likely to be endemic of this region, which has proven to be an area with high diversity and endemism in other small vertebrates (Ríos-Alvear & Reyes-Puig, 2015).

The collection sites consisted of mature Andean forest (Ecuadorian Ministry of the Environment (MAE), 2012) with emergent trees in a closed canopy and an understory of herbaceous vegetation and abundant leaf litter. The calls of *N. naturetrekii* were heard throughout the day in these sites. Likewise, the majority of individuals were obtained during the day in pitfall traps in Vizcaya during the month of March, indicating that it is mostly a diurnal species, a characteristic trait of the genus *Noblella* (Guayasamin & Terán-Valdez, 2009; Harvey et al., 2013). It is noteworthy that one of the specimens (DHMECN 13307) collected in the locality of Vizcaya was found in secondary forest, dominated by Andean bamboo (*Chusquea* sp). The entrance path to the Naturetrek Reserve in which it was found was destroyed a few weeks later for the opening of a new road.

The ZSFQ specimens were found on the forest floor hidden in the leaf litter. Two males were active at 22:00 in a forest patch next to the road. Both males were vocalizing within a dead trunk each at an approximate height of 50 cm above the ground, where there was abundant litter on the trunk, the female was found among litter on the ground. Other males were heard in the same locality at the same time. At this locality, the species is sympatric with *Gastrotheca testudinea*, *Pristimantis modipeplus*, and *Anolis orcesi*.

**Etymology.** *Noblella naturetrekii* is an emblematic species of the mosaic of Naturetrek Reserves, owned and managed by the Ecominga Foundation, which protect the cloud forests in the upper Pastaza River watershed. Naturetrek, a British wildlife tour operator, has fully funded the purchase of these forest reserves, in two of which the new frog species was discovered. These funds were transferred to the Ecominga Foundation via the World Land Trust. The name is the latinized possessive used in apposition to honor the efforts of the company Naturetrek.

## Discussion

According to our phylogeny, the genus *Noblella* is non-monophyletic. Solving the polyphyly of *Noblella* will require changing the genus of one of the two clades of *Noblella*. We refrain from proposing a new generic arrangement until the phylogenetic position of *N. peruviana* and *Psychrophrynella bagrecito* (i.e., the type species of the respective genus) is clarified. Without DNA sequences of these two species it is impossible to resolve the taxonomic uncertainty.

The discovery of new species in the upper basin of the Pastaza River have helped to maintain biodiversity monitored, mainly small vertebrates (Museo Ecuatoriano de Ciencias Naturales (MECN), 2013). Terrestrial-breeding frogs are a group with high endemism and very restricted distribution in this important conservation area (Reyes-Puig, Reyes-Puig & Yánez-Muñoz, 2013; Reyes-Puig et al., 2014). It is surprising that we continue to discover species in these relatively well-studied areas, in which there has been field and laboratory work during the last decade (Reyes-Puig et al., 2010, 2019; Museo Ecuatoriano de Ciencias Naturales (MECN), 2013). Further demonstrating the importance of continuing research in the under-sampled strata of the forest, especially the forest floor and the canopy, where small vertebrates can be hidden (Guayasamin et al., 2006; Yánez-Muñoz et al., 2018). The search for strategies to reduce impacts of deforestation and climate change on a local level are key for specific actions to preserve the biodiversity of these forests. In the case of *N. naturetrekii*, the support of the natural tourism company Naturetrek was of vital importance for the preservation of the ecological reserves where this species is currently taking refuge. We have witnessed how forest has been replaced by agriculture and other development outside of these protected areas (i.e., cattle raising, forest exploitation), and we are gradually working with the local communities to plan and take advantage of the natural environment using some type of community tourism for the sustainable enjoyment of these protected areas. These areas currently provide sources of water supply for the community of Vizcaya, and they provide habitat for several endangered species, including the spectacled bear (*Tremarctos ornatus*), mountain tapir (*Tapirus pinchaque*), cougar (*Puma concolor*) and other small endemic vertebrates (e.g., *Pristimantis modipeplus*, *Pristimantis pastazensis*) as well as many locally-endemic plant species (Salazar & Jost, 2012; Reyes-Puig, Reyes-Puig & Yánez-Muñoz, 2013; Reyes-Puig & Ríos-Alvear, 2013; Ríos-Alvear & Reyes-Puig, 2013).

## Conclusions

We provide morphological, osteological, genetic, and acoustic evidence that validate the description of a new species, *N. naturetrekii*. We include a well-supported phylogeny that highlights *Noblella* as a non-monophyletic genus with two distinctive clades. The discovery of this new species in the upper basin of the Pastaza River watershed demonstrates the importance of conserving the zone and illustrates the need for future studies to help understand the actual biodiversity of these Andean Trans-Amazonian forests. Ecominga and Naturetrek Reserves protect biodiversity that inhabits between two large National Parks. Therefore, strategies to maintain connectivity between these conservation areas can be addressed by the continuing description of new species.

## Supplemental Information

10.7717/peerj.7405/supp-1 Supplemental Information 1 Measurements of morphological characters of *Noblella naturetrekii* sp. n.

10.7717/peerj.7405/supp-2 Supplemental Information 2 Occurrence coordinates of *Noblella naturetrek* sp. n. The specific localities are included.

10.7717/peerj.7405/supp-3 Supplemental Information 3 Sequences matrix used for the phylogenetic analyses of *Noblella naturetrek* sp. n.

## Additional Information and Declarations

### Field Study Permissions

The following information was supplied relating to field study approvals (i.e., approving body and any reference numbers): Research was conducted under permission of the Dirección Nacional de Biodiversidad, Ministerio del Ambiente del Ecuador (N° 02-2018-IC-FAU-DPAT-VS, N° 01 2017-IC-FAU-DPAT-VS, N° 018-2017-IC-FAU-DNB/MAE).

### DNA Deposition

The following information was supplied regarding the deposition of DNA sequences: The sequences are available in GenBank: QCAZ 19764, *Craugastor longirostris*: (16S) MK838464, (RAG1) MN068026; QCAZ 45959, *Niceforonia* sp., (16S) MK838463, (RAG1) MN068027; QCAZ 58818, *Noblella personina*, (12S) MK838468, (16S) MK838465, (RAG1) MN068028; DHCM 13307, *Noblella naturetrekii* sp. n., (12S) MK838467, (16S) MK838462.

### Data Availability

The following information was supplied regarding data availability: The raw measurements of specimens and the occurrence points of the species are available in Supplemental Files.

### New Species Registration

The following information was supplied regarding the registration of a newly described species: Publication LSID: urn:lsid:zoobank.org:pub:3D663D4F-1AFF-4949-9C18-FFE37C97196A. Noblella LSID: urn:lsid:zoobank.org:act:9D326495-F91B-4B4A-A781-6FC78EDFBF43. Noblella naturetrekii sp. n. LSID: urn:lsid:zoobank.org:act:64223F8F-6FF2-4A45-B63D-1F0CE22C1113.
